# Forensic toxicological study on adipocere formation in submerged cadavers of female albino rats intoxicated with cadmium

**DOI:** 10.1080/20961790.2018.1541537

**Published:** 2019-01-31

**Authors:** Nabela I. El-Sharkawy, Yasmina M. Abd-Elhakim, Alklech M. Alklech

**Affiliations:** Department of Forensic Medicine and Toxicology, Faculty of Veterinary Medicine, Zagazig University, Zagazig, Egypt

**Keywords:** Forensic sciences, forensic toxicology, adipocere, cadmium, submersion, rats

## Abstract

There is a dearth of information on the mutual interaction between metal intoxication and adipocere formation. Herein, 40 adult female albino rats were distributed into two equal groups, one used as control while the other orally administered single dose of cadmium chloride (CdCl_2_) 225 mg/kg·bw (LD_min_). Control group was killed by cervical dislocation. Half of dead rats of both groups were subjected for determination of iodine value and estimation of cadmium (Cd) residues while the other half of both groups were submerged in opened glass container previously filled with 4 L dechlorinated tap water kept in closed room with an open air access (one cadaver/container). Gross morphological changes of submerged cadavers were recorded weekly along the experiment. At the end of the experiment, after 3 months, samples were collected again for iodine value determination and estimation of Cd residues. The obtained results revealed the depressant effect of Cd toxicity on development of adipocere. Cd residues were found in different tissues of cadavers at time of death with the highest amount in the intestines followed by the liver and kidneys, then lungs, adipose tissue, muscles, and finally the bones. After 3 months of water submersion, tissues exhibited significant decrease in the amount of Cd residues but to a limit that was still detected. This study concluded the possibility of detection of Cd residues even after adipocere formation. Additionally, it shed light on the possibility of the interference of environmental pollution with the natural rate of decomposition especially adipocere formation.

## Introduction

Forensic toxicology poses a great challenge to the toxicologist analyst. The variety of analytes is large, and the quantity and the quality of available material may be very limited especially from putrefied, mummified, adipoceratus, or severely burned bodies [1]. Adipocere formation may occur in any site whereby fatty tissue or lipids are found before death, including internal organs. Moreover, adipocere formation can occur in tissues with minimal fat content because of the translocation of liquefied fat and subsequent diffusion into the tissue [2]. In a forensic context, adipocere is important because of its ability to slow decomposition and, in some cases, preserve the remains [3–5]. Various poisons as cyanide, strychnine and arsenic have been reported to impede the decomposition processes and enhance adipocere formation [6].

Cadmium (Cd) is regarded as highly toxic by the Association of American Feed Control Officials Incorporation, in that its vehiculation is possible to animals through the ingestion of contaminated mineral formulations [7]. Cd is well known to be highly toxic to humans and animals [8]. Cd is one of the most abundant metals in our environment, with no biological function in superior organisms described so far, which is very toxic even at very low concentrations [9]. Routes of Cd exposures include gastrointestinal, pulmonary and dermal routes. The dermal route was doubtful until it was confirmed that the resorption of Cd by human cadaver skin in a diffusion cell model from Cd-contaminated soil [10].

Highly toxic concentrations of two heavy metals (Cd: 0.30 mg/kg; thallium: 0.91 mg/kg) and minor levels of three organic substances (phenobarbitone: 0.32 mg/kg; nordazepam: 0.14 mg/kg; salicylic acid: 0.04 mg/kg) were detected in adipoceratous samples in dead human body after a postmortem period of nearly 3 years despite advanced skeletization and complete transformation of the still existing residual soft tissues to adipocere [11]. In a recent study, Cd intoxication induced ocular alterations which retain the same trend in correlation with postmortem interval as natural deaths except for the retinal DNA damage [12].

While the formation of adipocere has been intensively investigated, both under laboratory [13–15] and real conditions [16,17], only a few reports concentrate on role of antemortem Cd exposure on adipocere formation. Thus this work is designed to investigate the effect of Cd toxicity on rate of adipocere development via tracing morphological changes in the submerged cadavers and determination of iodine value and estimation of Cd residues in both subcutaneous and visceral fat. The effect of adipocere on Cd residues in different tissues of intoxicated female albino rats is also evaluated.

## Materials and method

### Test compound and other reagents/chemicals

Cadmium chloride (CdCl_2_, 99.5% purity) was purchased from Alpha Chemica Mumbai, India. It was diluted to working stock concentrations for use in the experiment using de-ionized water. All other reagents/chemicals used were purchased from Sigma (St. Louis, MO, USA) and were of analytical grade.

### Animal grouping and experimental design

Forty Sprague Dawely albino rats (adult female, 8 week-of-age, and 225 g) were purchased from the Laboratory Animal Farm at Zagazig University. All rats were housed in stainless steel cages in a pathogen-free environment maintained at a controlled temperature (21 °C–24 °C) with a relative humidity of 50%–60% and a 12-h light-dark cycle. All rats had ad libitum access to standard rodent chow and filtered water throughout the study. Rats were acclimated 2 weeks prior to use in any study herein.

At the commencement of the experiment, rats were weighed and randomly distributed into two groups, each containing 20 rats. Group I (Control group) rats received 1 mL distilled water. Group II (Cd intoxicated group) rats were orally administered single dose of CdCl_2_ 225 mg/kg·bw (LD_min_) [18]. Rats of control group were killed by cervical dislocation. Half of dead rats of both groups were subjected for determination of iodine value and estimation of Cd residues while the other half of both groups were submerged in opened glass container previously filled with 4 L dechlorinated tap water kept in closed room with an open air access (one cadaver/container). The container was designed to allow insect scavenging and full access to climatic conditions, enabling the decomposition process to naturally occur. At the end of the experiment after 3 months, samples were collected again for iodine value determination and estimation of Cd residues.

### Observations

Along the experimental period, gross morphological changes of submerged cadavers were photographed weekly using a Nikon DX digital camera (AF-S DX NIKKOR; Melville, NY, USA), at 20 cm distance and with a resolution of 10.2 megapixels for the postmortem changes. Also, the degree of water clearance and ambient temperature was recorded.

### Determination of iodine value

Subcutaneous and visceral fat were collected from cadavers at the time of death and after 3 months of water submersion where isolation and purification of fat and iodine value determination were carried out [19,20].

### Estimation of Cd residues

#### Preparation of standard solutions, instruments and reagents

Using pure certified metals and Merck grade HNO_3_ (65%) and H_2_O_2_ (30%) (HPLC grade, Merck, Darmstadt, Germany), standard solutions were prepared at different µg/mL levels to construct a calibration curve. All plastic ware and glassware were cleaned by rinsing several times with distilled water, soaking in HNO_3_ (10%) and rinsing in de-ionized water prior to use. All reagents were of analytical grade. All working solutions were prepared with de-ionized water.

#### Digestion procedure

Tissue samples from liver, kidneys, stomach, intestines, adipose tissue, bones, lungs and muscles from both control and Cd intoxicated groups were kept frozen for residue analytical study and then subjected to acid digestion according to [21]. One gram of the sample was transferred to a clean screw-capped glass bottle and digested with 4 mL of digesting solution (*V* (65% HNO_3_):*V* (70% HClO_4_) = 1:1). Initial digestion was carried out for 24 h at room temperature (14 °C–24 °C), followed by heating at 110 °C for 2 h. After cooling redistilled water (or de-ionized water) was added, the solutions were warmed in water bath for 1 h to expel nitrous gases. Digests were then filtered (Whatman No. 1) and diluted to 25 mL de-ionized water [22]. The resultant solutions were then maintained in a refrigerator until the analyses were performed. The same procedure was applied to the blanks.

#### The analytical procedures quality assurance

A standard reference material (NBS-Bovine Liver, No.1577a) from the National Institute of Standards and Technology (NIST) was used to control the precision and the accuracy of the analytical procedure. Comparisons of the certified values for each metal with the analyses of the reference material revealed recovery rates of 98% for Cd respectively.

### Determination of Cd residues

Determination of Cd was conducted at the central laboratory, Faculty of Veterinary Medicine, Zagazig University, Egypt. A Buck Scientific 210 VPG Atomic Absorption Spectrophotometer, flame technique (FAAS), was used. The instrumental parameters for Cd were as following: 228.9 nm lamp wavelength, 2.0 mA lamp current, 0.7 nm slit width, 13.5 L/m air “support gas” flow, 2.0 L/m acetylene “fuel gas” flow, 4 cm burner height and limit of detection (LOD) = 0.05 µg/mL.

### Statistical analysis

Data were expressed as means ± SE. Evaluations of the results were performed using a one-way analysis of variance (ANOVA) followed by Duncan's Multiple Range test. Statistical analyses were performed using Software GRAPHPAD INSTAT (Graphpad Software, La Jolla, CA, USA). A *P*-value <0.05 was accepted as significant. In addition, a GraphPad computer programme was used to conduct regression analysis and to plot collected data. All data were tested for normality via a Shapiro-Wilk test and results reported to ≤0.05, level of significance.

## Results

### Changes in gross morphology of cadavers

**Figure 1.**
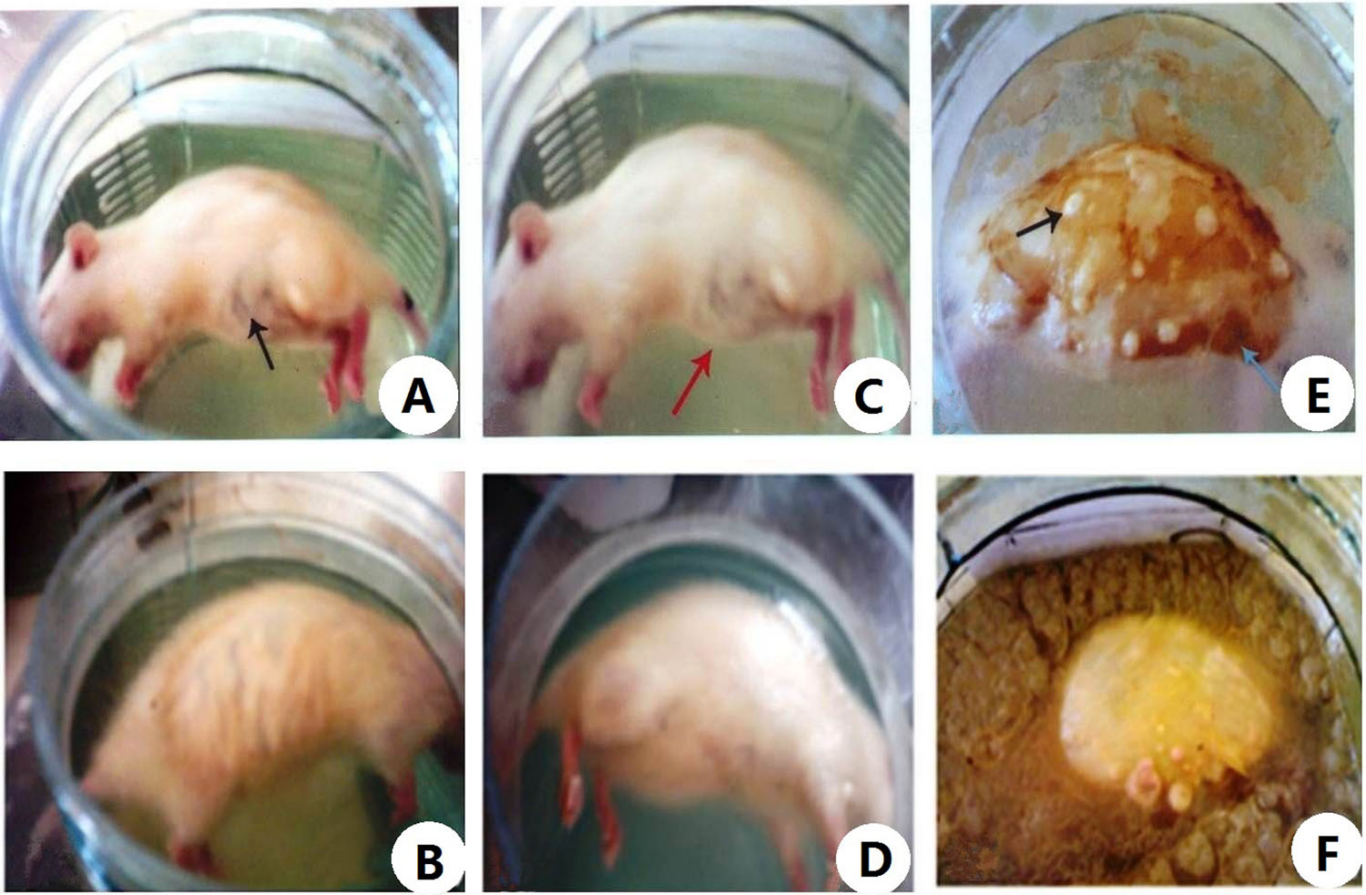
Postmortem changes of cadavers of control and cadmium chloride (CdCl_2_) intoxicated female albino rats submerged in water from 1st to 3th week of the experiment. (A) Showing cadaver of a female albino rat of control group at the end of the 1st week of water submersion, floated high on the water surface with exposure of lateral aspects of body and bloated with some greenish discolouration of the abdominal wall (black arrow). (B) Showing cadaver of a female albino rat intoxicated with single dose of CdCl_2_ 225 mg/kg·bw (LD_min_) at the end of the 1st week of water submersion, floated high on the water surface with exposure of lateral aspects of body without any noticeable changes. (C) Showing cadaver of a female albino rat of control group at the end of the 2nd week of water submersion, with some greenish discolouration along the thoracic and abdominal wall (red arrow). (D) Showing cadaver of a female albino rat intoxicated with single dose of CdCl_2_ 225 mg/kg·bw (LD_min_) at the end of the 2nd week of water submersion, with little bloating. (E) Showing cadaver of a female albino rat of control group at the end of the 3rd week of water submersion, with thin layer of brownish sediment deposited along the exposed parts of body (blue arrow) and numerous whitish fungal patches (black arrow). (F) Showing cadaver of a female albino rat intoxicated with single dose of CdCl_2_ 225 mg/kg·bw (LD_min_) at the end of the 3rd week of water submersion, with softening of the flesh and murky water.

**Figure 2.**
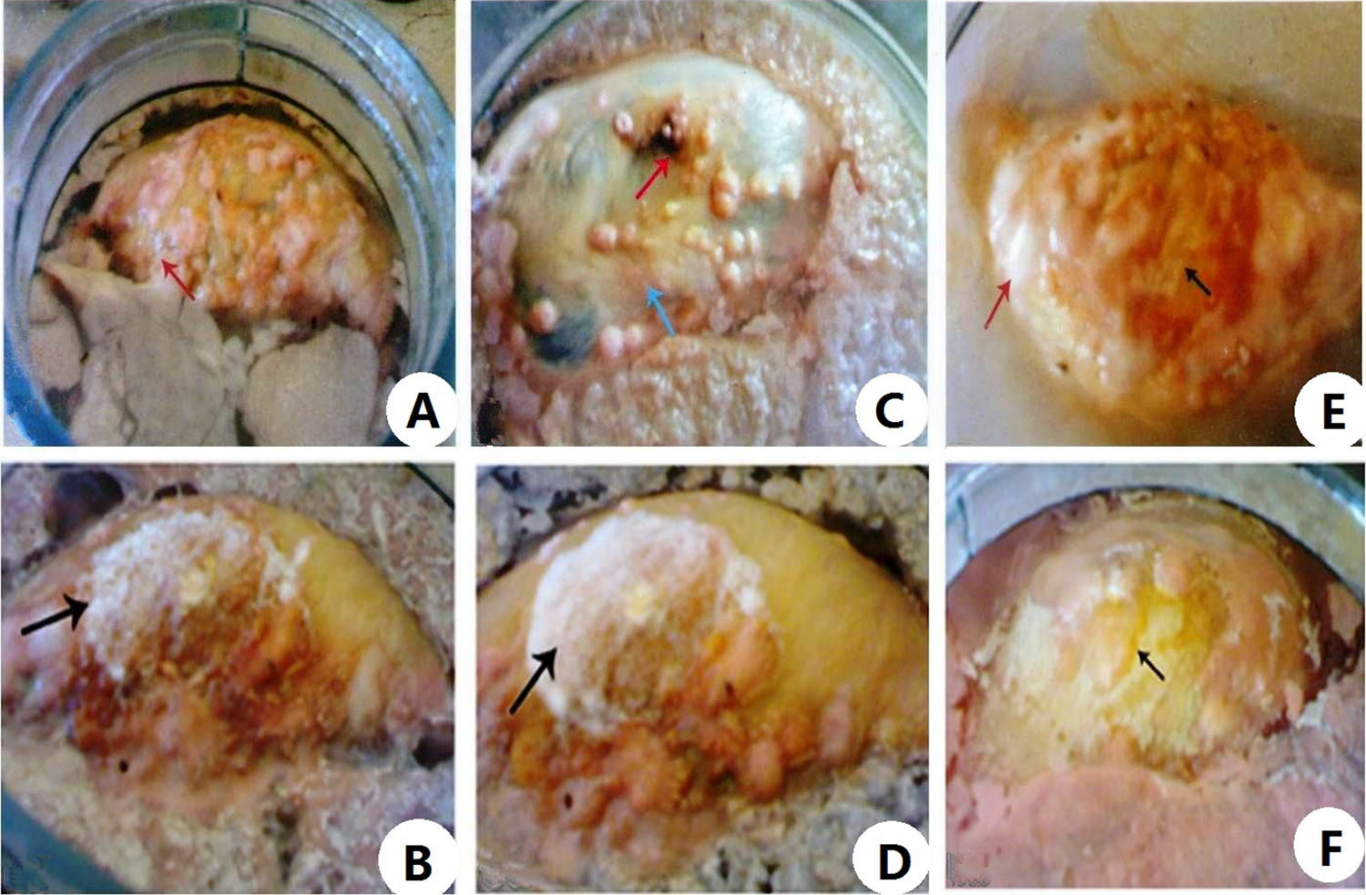
Postmortem changes of cadavers of control and cadmium chloride (CdCl_2_) intoxicated female albino rats submerged in water from 4th to 6th week of the experiment. (A) Showing cadaver of a female albino rat of control group at the end of the 4th week of water submersion, with light orange patches along the exposed parts of body (red arrow) and cloudy water. (B) Showing cadaver of a female albino rat intoxicated with single dose of CdCl_2_ 225 mg/kg·bw (LD_min_) at the end of the 4th week of water submersion, with extensive whitish patches (black arrow). (C) Showing cadaver of a female albino rat of control group at the end of the 5th week of water submersion, with brownish (red arrow) and light orange (blue arrow) patches along the exposed parts of body beside scum of fluid over the surface of cloudy water. (D) Showing cadaver of a female albino rat intoxicated with single dose of CdCl_2_ 225 mg/kg·bw (LD_min_) at the end of the 5th week of water submersion, with extensive whitish patches (black arrow). (E) Showing cadaver of a female albino rat of control group at the end of the 6th week of water submersion, the exposed parts of the body and those closer to the water level had changed from the original pigmentation to a pale yellow colour (black arrow) with whitish patches (red arrow). (F) Showing cadaver of a female albino rat intoxicated with single dose of CdCl_2_ 225 mg/kg·bw (LD_min_) at the end of the 6th week of water submersion, the exposed parts of the body and had changed to a pale yellow colour (black arrow).

**Figure 3.**
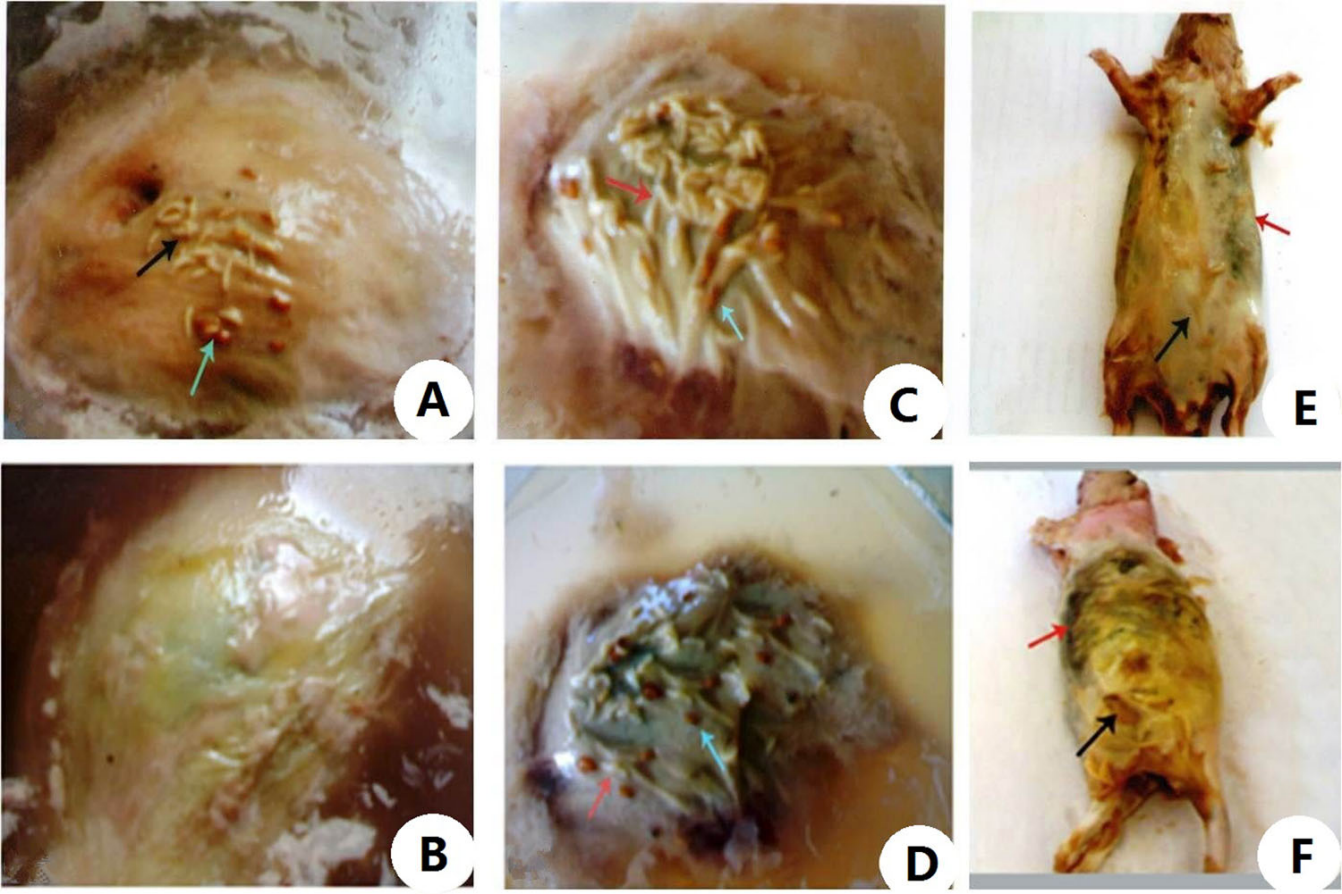
Postmortem changes of cadavers of control and cadmium chloride (CdCl_2_) intoxicated female albino rats submerged in water from 7th to 12th week of the experiment. (A) Showing cadaver of a female albino rat of control group at the end of the 7th week of water submersion, with some insect activity represented in presence of some larvae (black arrow) and pupa of flies (blue arrow). (B) Showing cadaver of a female albino rat intoxicated with single dose of CdCl_2_ 225 mg/kg·bw (LD_min_) at the end of the 7th week of water submersion, extensive pale yellow colouration of the exposed parts of the body. (C) Showing cadaver of a female albino rat of control group at the end of the 8th week of water submersion, with marked increase in larvae (red arrow) and pupa of flies (blue arrow). (D) Showing cadaver of a female albino rat intoxicated with single dose of CdCl_2_ 225 mg/kg·bw (LD_min_) at the end of the 8th week of water submersion, with presence of some larvae (red arrow) and pupa of flies (blue arrow). (E) Showing cadaver of a female albino rat of control group at the end of the 12th week of water submersion, with marked grayish white colouration especially at abdomen (black arrow) with greenish discolouration at the thoracic wall (red arrow). (F) Showing cadaver of a female albino rat intoxicated with single dose of CdCl_2_ 225 mg/kg·bw (LD_min_) at the end of the 12th week of water submersion, with grayish white colouration especially at abdomen (black arrow) with greenish discolouration at the thoracic wall (red arrow).

Table 1 and Figures 1–3 showed the external morphological changes of cadavers of female albino rats of the control and CdCl_2_ intoxicated group submerged in water and changes in surrounding environment. Following submersion cadavers of both groups floated on the water surface with exposure of the lateral side of body by the end of the 1st week there were bloating and greenish discolouration of the thoracic and abdominal wall which became highly marked in the control group by the end of the 2nd week in comparison of the control group. Along the next weeks there was progression of numerous coloured patches on the exposed parts of the cadavers and insect activity. By examination of the cadavers at the end of the experiment, there was generalized integrity of its parts with presence of whitish waxy greasy material on the abdominal wall that was so clear in control group compared to the Cd group.

**Table 1.** External morphological changes of cadavers of female albino rats submerged in water and changes in surrounding environment (water clearance and ambient temperature).

Submersion intervals	Groups	Changes							
		Floating of the cadaver	Bloating of the cadaver	Greenish discolouration of thoracic and abdominal wall of the cadaver	Coloured patches	Insect activity	Odor of decay	Water clearance	Ambient temperature (°C)
At the end of the 1st week	Control	Floating on water surface with exposure of lateral parts of cadaver	Moderate	Marked	–	–	Absent	Clear	19–21
	CdCl_2_ intoxicated		Little	Absent	–	–			
At the end of the 2nd week	Control	Floating on water surface with exposure of lateral parts of cadaver	Moderate	Marked	–	–	Absent	Still clear	21–24
	CdCl_2_ intoxicated		Little	Very faint	–	–			
At the end of the 3rd week	Control	Floating on water surface with exposure of lateral parts of cadavers	Maximum	Marked but covered with coloured patches	Numerous white patches on the exposed parts of cadavers	–	Pungent odor	Ranged from turbid to murky with reddish colouration	19–24
	CdCl_2_ intoxicated		Little	Very faint	Little white patches on the exposed parts of cadavers	–			
At the end of the 4th week	Control	Floating on water surface with exposure of lateral parts of cadavers	Maximum	Marked but covered with coloured patches	Numerous light orange patches on the exposed parts of cadavers	–	Offensive odor	Dense and cloudy with thin oily film on the surface	17–21
	CdCl_2_ intoxicated		Little	Very faint	Extensive white growth on the exposed parts of cadavers	–			
At the end of the 5th week	Control	Floating on water surface with exposure of lateral parts of cadavers	Maximum	Marked but covered with coloured patches	Scattered light orange and brownish patches on the exposed parts of cadavers	–	Very offensive odor	More cloudy with obvious scum on the surface	18–21
	CdCl_2_ intoxicated		Little	Very faint	Extensive white growth beside little orange one on the exposed parts of cadavers	–			
At the end of the 6th week	Control	Floating on water surface with exposure of lateral parts of cadavers	Maximum	The exposed parts of the body and those closer to the water level had changed a pale yellow colour	Slimy layer consisting of both light orange and whitish patches covered most of exposed areas of the cadavers	Some flies were noticed on the exposed surface of the cadavers	Very offensive odor	Total opacity	20–21
	CdCl_2_ intoxicated		Little						
At the end of the 7th week	Control	Floating on water surface with exposure of lateral parts of cadavers	Maximum	Extensive pale yellow colouration of the exposed areas	Insect activity covered coloured patches	Some insect activity represented in presence of some larvae and pupae of flies	Less prominent odor	Total opacity	16–17
	CdCl_2_ intoxicated		Little						
At the end of the 8th, 9th, 10th and 11th week	Control	Floating on water surface with exposure of lateral parts of cadavers	Maximum	The exposed surface ranged from yellow to whitish–gray The exposed surface ranged from yellow to whitish–gray	Increased insect activity covered layer	Presence of masses of maggots	Odor began to disappear	Opaque with of some falling hair and dead larvae on surface	14–16
	CdCl_2_ intoxicated		Little						
At the end of the 12th week (examination of cadavers outside water)	Control	Complete integrity of parts of cadaver where tissues above the “water-level” zone become dry and hardened while those below were soggy with no tissue loss or bone exposure. The hair was easily removed and underneath was tense skin. Homogenous, friable and grayish white waxy material was easily identified with removal of skin. Greenish discolouration of the thoracic and abdominal wall was so clear with removal of the hair. The internal organs were still recognized definitely. Less integrity of parts of cadavers but friable and grayish white material was identified with removal of skin.							15–17
	CdCl_2_ intoxicated								

### Measurement of iodine values of fat samples

As shown in Table 2, the iodine value of both subcutaneous and visceral fat recorded the highest level at time of death compared with those after 3 months of water submersion. At time of death, iodine value of both subcutaneous and visceral fat recorded the highest value in control group followed by CdCl_2_ intoxicated group. After 3 months of water submersion iodine value of both subcutaneous and visceral fat showed highly significant decrease in control and CdCl_2_ intoxicated groups, respectively.

**Table 2.** Iodine value of subcutaneous and visceral fat from cadavers of female albino rats of both groups at time of death "fresh" and 3 months post water submersion "adipoceratous".

Groups	Time of sampling	Iodine value (mean ± SE)	
		Subcutaneous fat (*n* = 4)	Visceral fat (*n* = 4)
Control	At time of death	79.3 ± 11.6^a^	60.2 ± 11.9^a^
	3 months post-submersion	5.2 ± 3.8	17.5 ± 5.5
CdCl_2_ intoxicated	At time of death	60.6 ± 17.1	54.2 ± 0.5
	3 months post-submersion	7.9 ± 3.3	20.1 ± 1.7

^a^*P*<0.05, compared with 3 months post-submersion.

### Measurement of Cd residues

Cadavers of female albino rats administered single oral dose of CdCl_2_ 225 mg/kg·bw (LD_min_) had different quantities of Cd residues in different tissues either at time of death or after 3 months of water submersion as represented in Table 3. The highest quantity of Cd residues was found in intestines: (100.8 ± 0.9) µg/g at time of death with significant decrease to (86.9 ± 0.8) µg/g after 3 months of water submersion followed by liver: (77.4 ± 1.0) µg/g with significant increase to (92.0 ± 1.4) µg/g after 3 months of water submersion. Kidneys behave in the same manner like liver with residues of (66.8 ± 0.8) µg/g with significant increase to (73.4 ± 0.8) µg/g after 3 months of water submersion.

**Table 3.** Cadmium residues (µg/g) in the different organs of cadavers of female albino rats administered single oral dose of CdCl_2_ 225 mg/kg·bw (LD_min_) at time of death and after 3 months of water submersion by atomic absorption spectrophotometer.

Time of sampling	Internal organs (mean ± SE, µg/g)						
	Intestines (*n* = 4)	Liver (*n* = 4)	Kidneys (*n* = 4)	Lungs (*n* = 4)	Fat (*n* = 4)	Muscles (*n* = 4)	Bones (*n* = 4)
At time of death	100.8 ± 0.9^a^	77.4 ± 1.0^a^	66.8 ± 0.8^a^	21.2 ± 0.6^a^	4.4 ± 0.3^a^	2.9 ± 8.1^a^	2.6 ± 0.1^a^
3 months post-submersion	86.9 ± 0.8	92.0 ± 1.4	73.4 ± 0.8	17.8 ± 0.6	2.6 ± 0.2	2.5 ± 9.5	2.7 ± 4.6

^a^*P*<0.05, compared with 3 months post-submersion.

Lungs of female albino rats administered single oral dose of CdCl_2_ 225 mg/kg·bw (LD_min_) showed residues of (21.2 ± 0.6) µg/g with significant decrease to (17.8 ± 0.6) µg/g after 3 months of water submersion. Both adipose tissue and muscles showed minimal Cd residues (4.4 ± 0.3) µg/g and (2.9 ± 8.1) µg/g at time of death with significant decrease to (2.6 ± 0.2) µg/g and (2.5 ± 9.5) µg/g, respectively after 3 months of water submersion. The least concentration of Cd residues of female albino rats administered single oral dose of CdCl_2_ 225 mg/kg·bw (LD_min_) was detected in bones: (2.6 ± 0.1) µg/g, and it was significantly increased after 3 months of water submersion.

## Discussion

An important objective of the postmortem forensic toxicological analysis is the identification of the toxic compound or certain poison in animal carcasses, tissues and body fluids for clarifying the cause of death especially in intended poisonings that can be punished as criminal activities or as dealing with cadavers passing through advanced state of decay such as adipocere [23,24].

In the present work, the early observable changes in the submerged cadavers were bloating and greenish discolouration of the thoracic and abdominal wall. This colouration is correlated with intestinal anaerobic bacteria, especially *Clostridium welchii* which break down hemoglobin and transfer it into sulfohemoglobin and other coloured pigments which extends from the right iliac fossa to the whole of the abdomen and thorax. These bacteria are also responsible for the formation of gases like sulphureted hydrogen, phosphoretted hydrogen, methane, carbon dioxide, ammonia and hydrogen and some mercaptans which are responsible for the detected unpleasant odor [24]. It was noticed that in the present study this greenish discolouration was so faint in CdCl_2_ intoxicated group compared with the control. This variation may be attributed to the destructive repressive effect of CdCl_2_ on most types of bacteria as bacterial surfaces are often negatively charged in natural environments and their surfaces display functional groups that effectively bind dissolved cationic metals like Cd^+2^ [25,26].

By the end of 3rd week of water submersion, we noticed presence of brownish colouration along the exposed surface of most of cadavers and reddish discolouration of the water with presence of scum on its surface which previously recorded by a study on submerged human cadaver by O'brien and Kuehner [27] who attributed brownish colouration of the cadaver to dryness of the exposed surface by air and reddish colouration of water to sloughed soft-tissues and fluids seeping from the cadavers especially from mouth and nostril under the effects of the pressure of putrefactive gases.

Our study revealed a progression of different coloured patches (white, bright orange and brownish) on the exposed parts of the cadavers from the 4th week of water submersion till the end of the experiment. The most notable one was the bright orange patches, which may belongs to *Fusarium* spp. that is commonly considered as a contaminant occasionally involved in skin and nail [28].

In the present work, the insect activity was noticed by the end of the 6th week. This finding disagrees with previous studies which reported that flies usually arrive at the corpse and lay eggs on it within 1 h or 2 h after death and takes 1–2 weeks till maturation [29,30]. The delayed insect activity noticed in the present work may be attributed firstly to kept of the jars containing cadavers in closed room with only one open access for air, secondly to covering of natural body orifices with water subsequently prohibit release of putrefactive gases which attract flies and lastly to the influence of climatic conditions whereas the study was conducted in winter season with faint insect activity [30].

It was noticed that all cadavers in the presented study were either floated on the water surface or partially submerged and never sinking throughout the experiment in accordance with observations given by O'brien and Kuehner [27]. Thus cadavers not followed the typical stages of underwater postmortem activity that has been observed in comparable studies such as: floating, sinking, putrefaction, subsequent refloatation, differential decomposition and the final sinking [3,31]. It was demonstrated that when the cadavers falls into or is located in water, the putrefaction usually takes a different route as the cadaver would float for a variable period of time depending on how long it takes for the lungs to fill with water and the air to evacuate the body's cavities; the body would then become totally immersed and sink [27].

By the end of the 12th week there was whitish homogenous greasy material more obviously in lower abdomen in both groups with lower extension in Cd intoxicated cadavers which is a characteristic feature to adipocere formation [32]. This phenomenon is attributed to hydrolysis and hydrogenation unsaturated fatty acid into saturated one.

Regarding determination of iodine value, it was noticed that the iodine value of both subcutaneous and visceral fat was higher in the control group than in the CdCl_2_ intoxicated group at time of death which may be associated with the initial presence of fat [33] and the effect of stress condition of intoxications. Additionally, it was noticed that the iodine value of the subcutaneous fat and visceral fat recorded the highest level at time of death compared with those after 3 months of water submersion as in agreement with Den Dooren De Jong, et al. [34]. This may be attributed to the diminishing of unsaturated fatty acid upon the microbial hydrogenation into saturated one [35]. Noteworthy, that iodine value decreased significantly in subcutaneous fat more than visceral fat which may be attributed to its relation of water in the surrounding environment [32].

Therefore based on the combined evidence of gross morphological changes of cadavers and chemical analyses of fatty tissues using iodine value, we could say that the cadavers of the two groups exhibited adipocere formation after 3 months of water submersion but showed significant variation in its degree with higher extension control cadavers than acutely intoxicated CdCl_2_ ones.

Noteworthy, in the present study adipocere is formed in ambient temperature ranged from 14 °C to 24 °C in agreement with the studies of O'brien and Kuehner [27] and Payne and King [31] dealing with submerged human and pig cadavers, respectively, as this may be a suitable temperature for growth of putrefactive micro-organisms involved in the process of adipocere formation.

Regarding residues of Cd at time of death in the present study, it was observed that intestines contained the highest amounts of Cd followed by liver, kidneys, lungs, adipose tissue, muscles and lastly bones. This finding agrees with those mentioned by Rikans and Yamano [36]. This may be attributed to retention of Cd by the intestinal mucosa until the intestinal cell was extruded into the intestinal lumen and the Cd eventually excreted [37]. The variation in the amounts of Cd remaining in the different tissues of the submerged cadavers at the end of the experiment after 3 months was quite striking as there was significant increase in the liver, kidneys and bone while those in intestines, lungs, adipose tissues and muscles were significantly decreased. An attractive clue for this site dependent difference could arise from an incomplete distribution of the poison at the time of death, and/or from postmortem redistribution at the cellular level by passive diffusion or via the vascular pathway from the major organs [38].
